# In this month’s Bulletin

**DOI:** 10.2471/BLT.19.000719

**Published:** 2019-07-01

In the editorial section this month, David Watkins (442) considers policy options for diet-related noncommunicable diseases. Peter Salama et al. (443) discuss the implications of research on health-worker strikes in low-countries.

Andrey Shukshin (446–447) reports on progress made in the Russian Federation to improve the treatment of childhood cancer. Sandro Galea talks to Gary Humphreys (448–449) about the power of epidemiology and the need to change the way we talk about health.

## Mexico

### How women deliver

Tarsicio Uribe-Leitz et al. (501–512) examine trends of caesarean delivery.

## Portugal

### Nutrition and deaths

Francisco Goiana-da-Silva et al. (450–459) model impacts of food industry co-regulation on noncommunicable diseases.

## South Africa 

### Ensuring the right to an identity

Nadine Nannan et al. (468–476) estimate completeness of birth registration.

## Global

### Ratio of women to men

Meghan A Bohren et al. (477–484) measure gender balance in WHO panels that develop guidelines.

### Working conditions in the health sector

Giuliano Russo et al. (460–467) gather the available evidence on health workers’ strikes in low-income countries.

### Extended-spectrum β-lactamase-producing Enterobacteriaceae

Yanhong Jessika Hu et al. (485–500) review antibiotic resistance data from infections in children in 20 countries.

